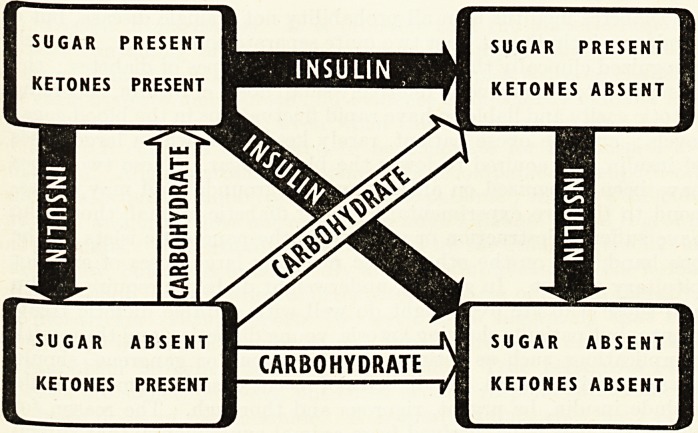# Some Points in the Treatment of Diabetes

**Published:** 1948

**Authors:** G. A. Smart

**Affiliations:** Bristol


### SOME POINTS IN THE TREATMENT OF DIABETES

BY

G. A. Smart, M.D., B.Sc., M.R.C.P.

*Bristol.*

---

*Diabetic Coma.* Patients who die of diabetes mellitus die in diabetic coma. This highly dangerous complication is preventable, therefore the first aim in treatment should be to prevent it. Although the cause of coma in diabetes is not known, the condition is closely correlated with ketosis, which almost invariably precedes its onset. The ketotic or pre-comatose state need never be fatal : whereas, however good the treatment, cases of true coma often die. Moreover, diabetic coma is rarely of acute onset ; at least twenty-four hours (and usually several days), elapse during which gross ketosis develops before the onset of loss of consciousness and circulatory collapse. The best insurance policy therefore in the prevention of diabetic coma is the regular testing of the urine for ketone bodies. This is of much greater importance than testing for sugar. It cannot be over-emphasized that hyperglycaemia itself is not in the short term harmful : safety from diabetic coma lies in ensuring freedom from ketosis and not in ensuring normal blood-sugar.

Ketosis arises whenever carbohydrate metabolism is abnormally low, either from the inadequate intake of carbohydrate or from insufficient or inefficient action of insulin or both. Ketosis is therefore treated by giving more insulin or by ensuring a greater carbohydrate intake or both.

It is essential that the patient be correctly instructed in this matter. Most patients have the firmly rooted idea that *sugar* in diabetes is harmful. If they test the urine for this substance only they may, in the pre-comatose state, note an excessive glycosuria, cut down their carbohydrate, and thus precipitate the onset of true and dangerous coma. I emphasize this point, because I am constantly having to treat patients in diabetic coma precipitated in this manner. If the patient is only capable of carrying out one kind of test on the urine let it be for ketones.

The classical tests for ketones (ferric chloride or Rothera's), are not suitable for patients.

Powder for Ketone Test.[1]

| | | |
|---|---|---|
| ℞ | Sodium nitro prusside .. .. .. | 0.5 per cent. |
| | Anhydrous tribasic sodium phosphate | 12.5 per cent. |
| | Ammonium sulphate .. .. .. | 87.0 per cent. |

Keep the powder dry in a well-corked bottle.

In a very simple test a small pile of powder is moistened with the urine and the colour read at the end of five minutes : this is well within the capacity of every patient. The test is very sensitive and instructions must be given that there is danger only when the purple colour is very deep, although with proper treatment this should never occur.

If the patient has not got a low renal threshold in addition to diabetes (and this combination is not unknown), and if the urine is tested both for sugar and ketones a simple scheme of treatment can be adopted.

The figure shows, in simplified form, four main types of results which may be obtained when the urine is tested. If ketones are present and sugar is absent, then carbohydrate metabolism is insufficient : the remedy is to increase the carbohydrate in the diet. If sugar is present with or without ketones more insulin should be given. Diabetics taking insulin are better with a slight glycosuria (shown by Benedict's test), since they are then less liable to have hypoglycaemic attacks. About 50 per cent. of diabetic patients are capable of understanding this scheme : it enables them to become self-reliant and to alter their dosage of insulin when this becomes necessary without unduly frequent reference to their medical adviser. The most important advice to those who cannot follow the scheme is that they must test the urine every morning for ketones and report at once if the result is positive.

*Insulin or Restricted Diet?* This focusing of attention on ketonuria rather than on glycosuria is necessary whether the diabetic is treated with insulin or not. Ketosis is always a danger signal and active steps should be taken to combat it. Nevertheless, many diabetics do not exhibit ketosis when first seen, the glycosuria apparently producing the main symptoms. Greater improvement may be obtained in such patients by a restricted diet and by measures to increase the utilisation of carbohydrate than by small doses of insulin. Incongruous as it may seem, when such cases are treated with insulin they frequently require larger doses than those who, without insulin, would rapidly develop ketosis and die. The requirement of insulin for stabilisation has no correlation with the severity of the diabetes.

Diabetes mellitus is in all probability not a single disease, but a syndrome including at least two quite separate conditions. It is well recognized clinically that there are two main types of diabetes : the thin patient, responsive to relatively small doses of insulin, becoming ketotic easily and liable to have rapid fluctuations in the blood-sugar levels ; and the obese patient, rarely ketotic, in whom large doses of insulin are required to lower the blood sugar. These two types have been recognized on anthropometric grounds[2] and may correspond to the two experimental types of diabetic animal, those who have suffered destruction or removal of the pancreatic islets on the one hand, and on the other those receiving large doses of anterior pituitary extract. In general, underweight diabetics require insulin and those who are overweight do well with suitable dietetic treatment : but patients showing ketosis, young diabetics, and those with complications such as retinopathy, infections or gangrene, should always receive insulin. In diabetes of recent onset treatment should include insulin, be urgent, rigorous and thorough. The reason for this is the new hope, derived from animal experiments, that sufficiently early and thorough treatment may cure the diabetic state.

*Prevention of Diabetic Gangrene.* In the older diabetic the most serious complication, apart from coma, is gangrene. This occurs almost invariably in the feet, danger points being toe-nails and callosities. There is no unequivocal evidence concerning the effect of various diets or of a high blood sugar on the development of arteriosclerosis in diabetes, but what evidence there is suggests that a diet high in carbohydrate is least likely to hasten its onset. Lawrence[3] has pointed out that patients with haemo-chromat osis are not especially liable to arteriosclerosis. Possibly poor control of diabetes contributes towards the development of arteriosclerosis, or perhaps both diabetes and arteriosclerosis are due to the same underlying causes. At all events, in the present state of our knowledge, it is wise to regard any irregularity in the control of diabetes as contributory towards the advance of arteriosclerosis. Diabetic gangrene may also in part be due to the presence of peripheral neuritis should this involve autonomic fibres controlling peripheral blood-flow.

To lessen the risks of gangrene developing, the diabetes should be strictly controlled, the feet should be kept perfectly clean, shoes should be chosen for fit, not fashion, and regular attendance should be made on the chiropodist. Injuries to the feet even of the most minor character should be avoided, and should be carefully treated if they do occur. Particular warning should be given to patients that they should never use uncovered hot-water bottles, since if peripheral neuritis be present pain-sensation may be diminished with a consequent risk of burns.

*Dietetic Requirements.* It is probable that the diabetic requires a diet containing more of the vitamin B complex than a normal person. Many members of the vitamin B complex act as co-enzymes in carbohydrate metabolism and evidence of deficiency is found not infrequently in diabetics, even when the diet contains amounts of vitamin B complex considered to be adequate for the normal individual. Angular stomatitis, sometimes accompanied by superficial glossitis, is quite common among diabetic patients ; it is very difficult to treat and often does not respond to large amounts of the B group of vitamins. It is usually considered to be the result of riboflavin deficiency, but this is undoubtedly an over-simplification. The lesion may develop or grow worse shortly after the start of insulin therapy. Sydenstricker (1939)[4] reports two such cases suggesting that the deficiency is made apparent by the rapid increase in carbohydrate metabolism brought about by the insulin. True pellagrous skin lesions are sometimes seen, but these usually respond satisfactorily to large doses of nicotinamide. A B-complex deficiency may play a part in the peripheral neuritis so common in diabetics, and cases have been reported in which the peripheral neuritis has developed rapidly after the start of insulin therapy. In general, however, peripheral neuritis is more common among diabetics who have had inadequate therapy over long periods.

It goes without saying that, in poorly controlled diabetes, when amino-acids are being converted in large quantities into carbohydrate there is likely to be some upset in protein metabolism. Rarely cases are seen in which there is a generalized oedema associated with low plasma-protein levels, but usually there is no gross deviation from the normal. It may be that the proportions of constituent plasma proteins may be upset, although the total level is normal. This has been found to be particularly marked in subjects with diabetic retinopathy and it was alleged that, where normal proportions could be obtained by feeding high protein diets, the retinopathy showed a remarkable improvement. There is sufficient evidence that diabetics should ensure that their diet is rich in vitamins of the B complex and that it contains adequate amounts of first-class protein.

The diet of diabetics receiving insulin should be as nearly normal as possible, but it should contain a larger proportion of the protective foods than usual. There has been much controversy over the relative values of high-fat and high-carbohydrate diets, equally good results being claimed by both schools. Diabetics taking insulin and feeling well frequently do not keep very carefully to a strictly prescribed diet. Provided insulin is used there seems to be no virtue in restricting the patient from eating just what he likes—including sugar in tea, if desired! It is alleged that more insulin is required under these conditions; but an injection is equally inconvenient whether it contains fifty or sixty units and one has to weigh the discomfort of dietetic restrictions against the alleged wastage of a few units of insulin; furthermore, an increase in the carbohydrate content of the diet does not necessarily increase the insulin requirements. If the patient is allowed a free choice of diet, however, it is essential he be warned to take similar amounts of food at the same meals from day to day. Widdowson has shown that there are normally large daily variations in food intake, and it would be difficult to ensure proper stabilization under these conditions.

*The Sort of Insulin.* The insulins in common use can be characterized by their length of action. Soluble insulin begins to act in about thirty minutes and continues for about six hours; globin insulin takes slightly longer to begin acting and continues for approximately twenty-four hours; zinc protamine insulin goes on acting up to thirty-six hours. These differences are due to the various rates at which true insulin is released from the site of injection. It will be seen that an injection of soluble insulin should be followed by a meal containing carbohydrate and the general scheme is to have an injection of insulin morning and evening, each followed by a good meal. In this way most of the daily food intake occurs at breakfast and supper. With globin or zinc protamine insulin, on the other hand, it is necessary to spread the food as evenly as possible throughout the day. Thus, something should be taken at breakfast, between breakfast and dinner, at dinner, at tea, at supper, and before going to bed at night. If this is not inconvenient the advantage of only one injection per day points to the use of globin or zinc protamine insulin. Zinc protamine insulin at times raises red painful lumps at the sites of injection. This can sometimes be overcome by changing the make: but if not globin insulin should be used, since this rarely gives rise to such difficulty. Patients who are unstable and therefore very difficult to control sometimes do well on two daily injections of globin insulin, one before breakfast and the other before supper.

*Summary.* The most important single aim in the treatment of diabetes is to keep the patient free from ketones. This can only be achieved by an adequate intake of carbohydrate, of which the metabolism is ensured by sufficient insulin. It may or may not be necessary to supplement the patient's own insulin by injections. The dose of insulin required is not necessarily correlated with the severity of the diabetes. For those patients requiring insulin the diet should be adequate for normal growth and activity and should be as nearly normal as possible, provided similar amounts are taken at the same meals, day by day, and a good intake of first-class protein and vitamin B complex is ensured.

Care of the feet is extremely important for all diabetics over forty.

The choice of the type of insulin for ordinary everyday use depends largely upon the daily feeding pattern of the patient.

### REFERENCES

[1] G. A. Smart and C. H. Darling, *Lancet* 1948, ii, 630.

[2] R. W. Schnieder, Lena A. Lewis and E. Perry McCullagh, *Amercan Journal Med. Science*, 1946, ccxii, 462.

[3] R. D. Lawrence, *Physician's Bulletin*, 1947, xii, 34.

[4] V. P. Sydenstricker, L. E. Geeslin and J. W. Weaver, *J.A.M.A.*, 1939, cxiii, 2137.

*I am indebted to the Bristol Royal Hospital for permission to use the illustration on p.* 77.—G. A. S.

## Figures and Tables

**Figure f1:**